# Extracellular cGMP Modulates Learning Biphasically by Modulating Glycine Receptors, CaMKII and Glutamate-Nitric Oxide-cGMP Pathway

**DOI:** 10.1038/srep33124

**Published:** 2016-09-16

**Authors:** Andrea Cabrera-Pastor, Michele Malaguarnera, Lucas Taoro-Gonzalez, Marta Llansola, Vicente Felipo

**Affiliations:** 1 Laboratorio de Neurobiología, Centro Investigación Príncipe Felipe de Valencia, Spain

## Abstract

It has been proposed that extracellular cGMP modulates the ability to learn a Y maze task, but the underlying mechanisms remained unknown. Here we show that extracellular cGMP, at physiological concentrations, modulates learning in the Y maze in a biphasic way by modulating the glutamate-nitric oxide-cGMP pathway in cerebellum. Extracellular cGMP reduces glycine receptors activation inducing a voltage-dependent calcium-channels-mediated increase of calcium in Purkinje neurons. This calcium increase modulates CaMKII phosphorylation in a biphasic way. When basal calcium concentration is low extracellular cGMP reduces CaMKII phosphorylation, increasing nitric oxide synthase activity, the glutamate-NO-cGMP pathway function and learning ability. When basal calcium is normal extracellular cGMP increases CaMKII phosphorylation, reducing nitric oxide synthase activity, the pathway function and learning. These data unveil new mechanisms modulating learning in the Y maze and likely other learning types which may be therapeutic targets to improve learning in pathological situations associated with altered cGMP levels.

Different forms of learning and memory are modulated by different mechanisms. There are multiple learning and memory systems in the mammalian brain (hippocampus, amigdala, dorsal striatum) which interact in different ways to control behavior^1^,^2^. Cerebellum also modulates some types of learning, such as motor, emotional and cognitive associative learning^3^. Cerebellum also modulates cognitive components of spatial learning^4^, visuo-spatial attention, working memory, motor learning, and verbal function^5^,^6^.

There is a large body of evidence indicating that synaptic plasticity modulates learning and memory. Alterations in synaptic plasticity underlie impairment of learning and memory in many situations^7^,^8^,^9^. N-methyl-D-aspartate (NMDA) receptors are critical for the rapid regulation of synaptic plasticity and of learning and memory^10^,^11^,^12^,^13^.

A main pathway mediating modulation of learning by NMDA receptors is the glutamate-nitric oxide (NO)-cyclic GMP (cGMP) pathway. Activation of NMDA receptors increases calcium which binds to calmodulin and activates neuronal nitric oxide synthase (NOS). This increases NO, which activates soluble guanylate cyclase, increasing cGMP. Part of the cGMP is released to the extracellular fluid through ATP-dependent transporters^14^

This glutamate-NO-cGMP pathway in hippocampus modulates some forms of learning and memory, including inhibitory avoidance learning^15^,^16^, spatial learning in the Morris water maze^17^ and object recognition^18^,^19^.

The glutamate-NO-cGMP pathway in cerebellum modulates the ability to learn a conditional discrimination task in a Y maze^20^,^21^,^22^,^23^,^24^,^25^,^26^. The function of this pathway in cerebellum and the ability to learn this Y maze task decrease in parallel during ageing, following developmental exposure to PCBs or methylmercury or chronic exposure to 2,5-hexanedione and in rat models of chronic hyperammonemia or hepatic encephalopathy^21^,^22^,^23^,^24^,^25^,^27^,^28^. The function of the glutamate-NO-cGMP pathway and learning ability increase in parallel in rats developmentally exposed to polybrominated diphenyl eter (PBDE99)^26^.

Patients with liver cirrhosis may present hepatic encephalopathy (HE) with cognitive impairment and motor alterations^29^. Ammonia is not properly detoxified in these patients, leading to chronic moderate hyperammonemia, which is a main contributor to the neurological alterations in HE^29^,^30^. A main animal model of chronic hyperammonemia used to study the mechanisms leading to cognitive and motor alterations is rats fed an ammonium containing diet^31^. These rats show chronic moderate hyperammonemia similar to that present in cirrhotic patients and reproduce some of the cognitive and motor alterations present in patients with minimal HE^21^,^29^. Hyperammonemic rats show impaired function of the glutamate-NO-cGMP pathway, reduced levels of extracellular cGMP in cerebellum and reduced ability to learn the Y maze task^21^.

Treatments that increase cGMP in cerebellum restore learning in rats with hyperammonemia or hepatic encephalopathy^29^. This may be achieved by administering phosphodiesterase inhibitors such as zaprinast or sildenafil^21^,^22^, anti-inflammatories as ibuprofen or inhibitors of MAP-kinase p38^23^,^32^, or compounds reducing GABA_A_ receptors activation such as bicuculline, pregnenolone sulfate or GR3027^33^,^34^,^35^. These data support that the glutamate-NO-cGMP pathway and formation of cGMP in cerebellum modulates the ability to learn this Y maze task. Moreover, Erceg *et al.*^21^ showed that increasing extracellular cGMP in cerebellum of hyperammonemic rats by continuous intracerebral administration through osmotic mini-pumps restores the ability to learn the Y maze task. As cGMP does not cross the cell membrane, this indicates that extracellular cGMP modulates the ability to learn the Y maze task.

Based on the above data we hypothesized that: (a) extracellular cGMP would modulate the ability to learn the Y maze task by modulating the glutamate-NO-cGMP pathway; (b) this modulation would be biphasic: increasing cGMP concentration until a certain “optimal” level will enhance the function of the pathway while further increase would reduce the function of the pathway. These hypotheses imply that extracellular cGMP would modulate the glutamate-NO-cGMP pathway inside the neurons by acting on some membrane protein.

The aims of this work were: 1) assess whether extracellular cGMP modulates the function of the glutamate-NO-cGMP pathway; 2) assess whether this modulation is biphasic; 3) assess if the extracellular cGMP concentrations optimal for the function of the pathway and for learning are in the same range; 4) identify the membrane molecule sensing extracellular cGMP and transducing the signal to the intracellular pathway; 5) analyse intracellular steps of the pathway modulated by extracellular cGMP.

The studies were performed in control and hyperammonemic rats as two models with different levels of extracellular cGMP and function of the glutamate-NO-cGMP pathway. This should facilitate testing the above hypotheses. The function of the pathway as a whole was analysed *in vivo* by microdialysis in freely moving rats by measuring NMDA-induced increase in extracellular cGMP. The steps of the pathway modulated by cGMP were analysed in freshly isolated cerebellar slices from control and hyperammonemic rats.

## Results

### Chronic intracerebral administration of extracellular cGMP normalizes the function of the glutamate-NO-cGMP pathway in hyperammonemic rats *in vivo*

**Figure 1:**
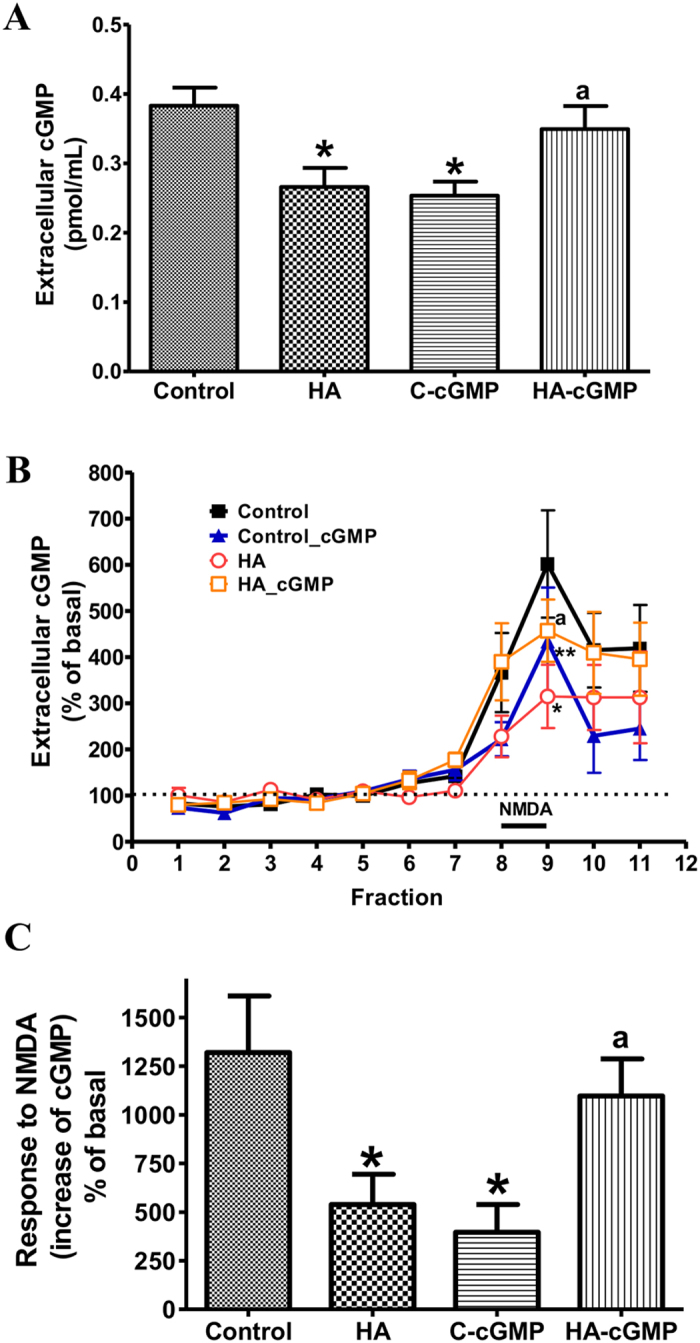
Chronic intracerebral administration of extracellular cGMP normalizes the function of the glutamate-NO-cGMP pathway in hyperammonemic rats *in vivo.* (**A**) Effect of chronic hyperammonemia (HA) and of chronic intracerebral administration of extracellular cGMP through osmotic pumps on basal levels of extracellular cGMP. (**B**) Time course of the effects of administering NMDA through the microdialysis probe on extracellular cGMP. Data are presented as percentage of basal values (mean of fractions 1–7). (**C**) Response to NMDA: NMDA-induced increase of extracellular cGMP. Values represent the accumulated increase in fractions 8–11 and are the mean ± SEM of 10 rats per group for controls and 8 rats per groups for hyperammonemic rats. Values significantly different from basal are indicated by “a” (p < 0.05). Values significantly different from control rats are indicated by asterisks, *(p < 0.05), **(p < 0.01).

In hyperammonemic rats extracellular cGMP in cerebellum (0.26 ± 0.02 nM) was lower (p < 0.05) than in control rats (0.38 ± 0.02 nM). Chronic administration of extracellular cGMP through osmotic mini-pumps normalized extracellular cGMP in hyperammonemic rats (0.35 ± 0.03 nM) and reduced it in control rats (0.25 ± 0.02 nM, p < 0.05) (Fig. 1A).

**Figure 2:**
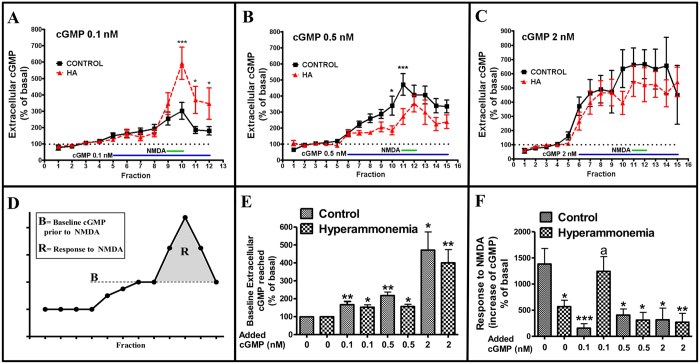
Effects of acute administration of different cGMP concentrations through the microdialysis probe on extracellular cGMP levels. Microdialysis was performed in cerebellum *in vivo* in control or hyperammonemic (HA) rats. (**A–C**) Effects of administration of 0.1 (**A**), 0.5 (**B**) or 2 (**C**) nM cGMP. Blue lines indicate application of extracellular cGMP and green lines application of NMDA. Dotted lines indicate basal levels. (**D**) Scheme showing what we mean with “baseline levels of cGMP prior to NMDA” and “response to NMDA”: the increase in cGMP induced by NMDA: (**E**) Shows the baseline levels of cGMP reached prior to addition of NMDA in A–C. Values are the mean ± SEM of fractions 5–8 for 0.1 nM and 6–10 for 0.5 and 2 nM cGMP. Data are presented as percentage of basal values (mean of fractions 1–4 in A and of fractions 1–5 in B and C). (**F**) Response to NMDA after administration of 0, 0.1 (**A**), 0.5 (**B**) or 2 (**C**) nM exogenous cGMP. Values are the mean ± SEM of 7 rats per group. Values significantly different from basal in hyperammonemic rats are indicated by a (p < 0.05). Values significantly different from basal in control rats are indicated by asterisks, *(p < 0.05), **(p < 0.01), ***(p < 0.001).

Figure 1B shows the time-course of extracellular cGMP levels prior to and following NMDA administration. From now on we will use the term “response to NMDA” as the increase in cGMP induced by NMDA (Fig. 2D). The response to NMDA was lower (p < 0.05) in hyperammonemic (539 ± 155%) than in control rats (1320 ± 290%). Chronic intracerebral administration of extracellular cGMP rescued the response to NMDA in hyperammonemic rats (1096 ± 191%, p < 0.05) and reduced it in control rats (396 ± 142%, p < 0.05) (Fig. 1C).

### Biphasic modulation of the glutamate-NO-cGMP pathway by acute administration of exogenous cGMP

Exogenous cGMP was administered at increasing doses (0.1, 0.5 or 2 nM) prior to and during stimulation with NMDA (Fig. 2A–C). Figure 2D shows an scheme of what we denominate “the baseline levels” of cGMP reached prior to NMDA administration and the “response to NMDA”. The baseline levels reached in control and hyperammonemic rats following administration of 0.1, 0.5 or 2 nM cGMP are shown in Fig. 2E.

Baseline cGMP levels modify the response to NMDA in a biphasic manner such that exogenous administration of cGMP in the control group inhibits the response to NMDA while in the hyperammnemic rats low levels (0.1 M) of cGMP significantly improved the response to NMDA while higher concentrations were inhibitory, suggesting biphasic modulation (Fig. 2F).

**Figure 3:**
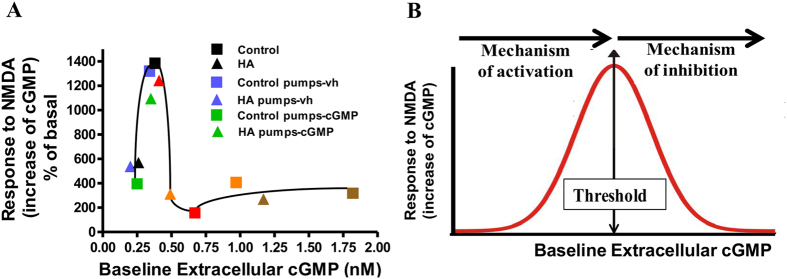
Extracellular cGMP modulates NMDA-induced activation of the glutamate-NO-cGMP pathway in a biphasic way. Data are from the same experiments shown in Fig. 2. The extracellular cGMP reached before addition of NMDA is expressed in nM. **(A)** Shows the response to NMDA: the increase in cGMP induced by NMDA as a function of baseline levels of cGMP (in nM) reached prior to NMDA addition. Control rats are indicated with a square □, hyperammonemic rats with a triangle ∆. The different colors indicate the concentration of exogenous cGMP perfused through the microdialysis probe: black, 0 nM; red, 0.1 nM; orange, 0.5 nM; brown, 2 nM; and cGMP perfused through pumps: blue, pumps with vehicle; green, pumps with cGMP. (**B**) A scheme of the biphasic modulation of the glutamate-NO-cGMP pathway function depending on the extracellular cGMP concentration.

Figure 3A shows the response to NMDA as a function of the baseline levels of extracellular cGMP reached in the experiments shown in Fig. 2. The data from chronic administration of cGMP with osmotic pumps (Fig. 1C) fit well in this curve, further supporting a biphasic modulation of the pathway by extracellular cGMP (Fig. 3A). The response to NMDA is maximal for a range of baseline levels of extracellular cGMP of approximately 0.30–0.45 nM. Lower or higher cGMP concentrations result in reduced function of the pathway.

Depending on the baseline levels reached, extracellular cGMP induced two different intracellular effects. Up to around 0.45 nM, extracellular cGMP induces an activating mechanism that enhances the glutamate-NO-cGMP pathway function. At concentrations higher than 0.45 nM, extracellular cGMP induces an inhibitory mechanism that reduces the pathway function (Fig. 3B).

### Extracellular cGMP modulates intracellular NOS and Ca^2+^/calmodulin-dependent protein kinase II (CaMKII) activities in cerebellar slices

**Figure 4:**
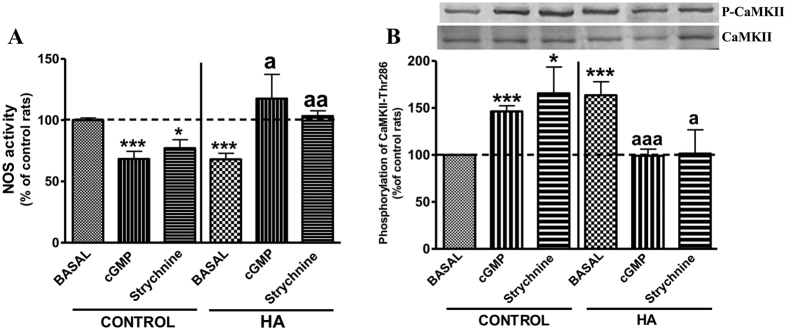
Effects of extracellular cGMP and strychnine on NOS activity (A) and CaMKII (B) phosphorylation in cerebellar slices. Cerebellar slices from control or hyperammonemic (HA) rats were incubated with 10 nM cGMP or 75 μM strychnine. NOS activity was determined by measuring conversion of radioactive arginine to citrulline and CaMKII phosphorylation was analyzed by western blot. A representative image of the blots is shown in (**B**). Values are the mean ± SEM of 16 rats per group. Data are expressed as percentage of basal values in control rats. Values significantly different from basal values in hyperammonemic rats are indicated by a (p < 0.05), aa (p < 0.01), aaa (p < 0.001). Values significantly different from basal values in control rats are indicated by asterisks, *(p < 0.05), ***(p < 0.001).

Basal NOS activity was lower (68 ± 5%, p < 0.001) in hyperammonemic than in control rats (Fig. 4A). Extracellular cGMP increased NOS activity in hyperammonemic rats to levels similar to control rats (118 ± 20% of controls, p < 0.05) and decreased NOS activity (p < 0.001) in control rats to 69 ± 6% of basal (Fig. 4A).

CaMKII activity was analyzed by measuring its phosphorylation in threonine-286 (Thr286). CaMKII phosphorylation was higher (164 ± 14%, p < 0.001) in slices from hyperammonemic than from control rats (Fig. 4B). Extracellular cGMP reduced CaMKII phosphorylation in hyperammonemic rats to normal levels (99 ± 7% of controls, p < 0.001) and increased it (p < 0.001) in control rats to 146 ± 6% of basal (Fig. 4B).

### Extracellular cGMP modulates the glutamate-NO-cGMP pathway by modulating glycine receptor

**Figure 5:**
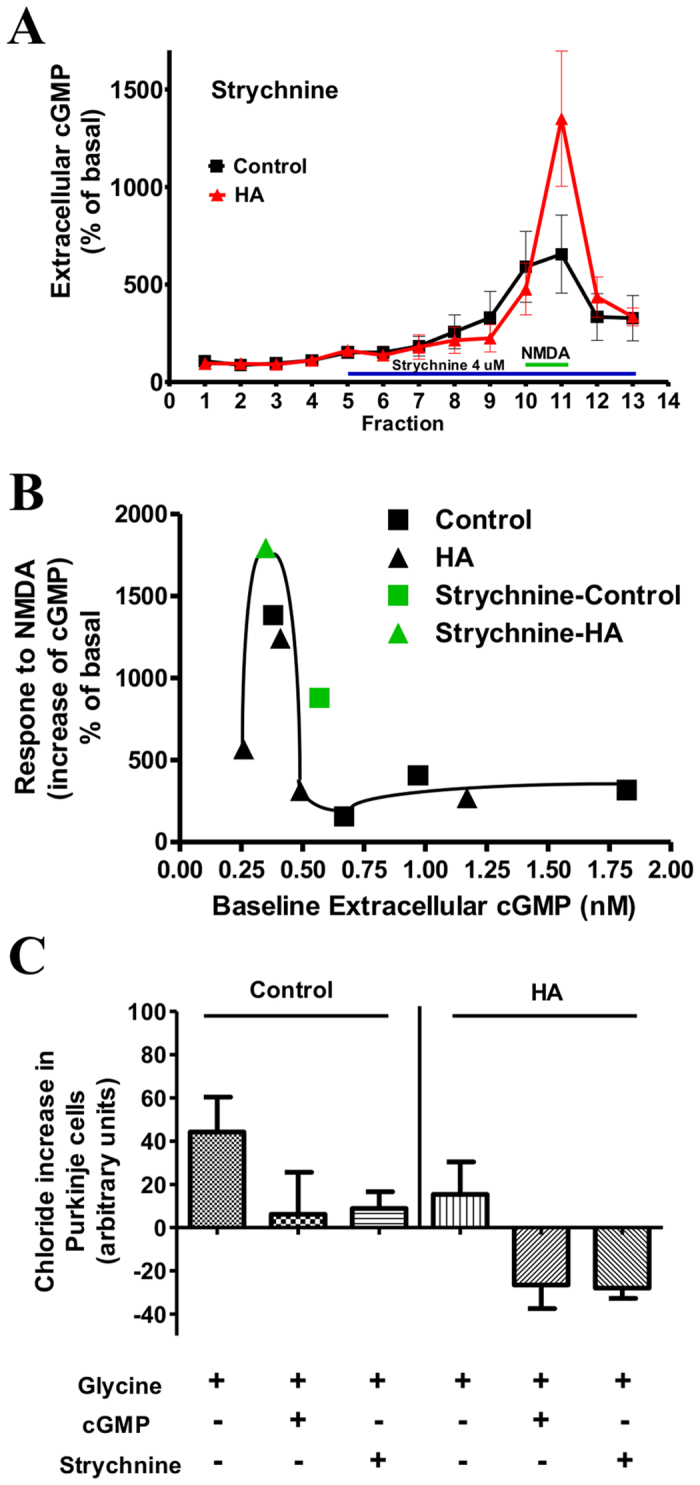
Effects of the glycine receptor antagonist strychnine on activation of the glutamate-NO-cGMP pathway *in vivo* and on intracellular chloride in slices. **(A)** Administration of strychnine (4 μM) through the microdialysis probe *in vivo*. Blue lines indicate application of strychnine and green lines application of NMDA. Data are presented as percentage of basal values (mean of fractions 1–4) of 6 rats per group. **(B)** Shows the same curve shown in Fig. 3A to which the data from (A) have been added in green color. Control rats are indicated with a square □, hyperammonemic rats with a triangle ∆. **(C)** Chloride increase in Purkinje cells from cerebellum slices of control rats, after administration of glycine (20 mM) alone or in combination with cGMP (10 nM) or strychnine (75 μM). Values are the mean ± SEM of at least 180 Purkinje neurons in at least 6 different slices. Values are the decrease in fluorescence (increase chloride) expressed as arbitrary units.

Adding strychnine (antagonist of glycine receptors) through the microdialysis probe modulates the glutamate-NO-cGMP pathway in a similar way to 0.1 nM cGMP: reduces the response to NMDA in control rats to 879 ± 363% and increases it in hyperammonemic rats to 1794 ± 589% (Fig. 5A). When these data were superimposed in Fig. 3A, they fit well in the curve, further supporting a biphasic modulation of the pathway (Fig. 5B).

Strychnine also modulates CaMKII phosphorylation and NOS activity in cerebellar slices in a similar way to cGMP. In control rats strychnine increased CaMKII phosphorylation to 166 ± 28% of basal (Fig. 4B) and reduced NOS activity to 77 ± 7% of basal (Fig. 4A). In hyperammonemic rats strychnine reduced CaMKII phosphorylation (101 ± 25% of basal in controls) (Fig. 4B) and increased NOS activity (103 ± 5% of basal in controls) to normal levels (Fig. 4A).

### Extracellular cGMP modulates intracellular chloride and calcium levels through modulation of glycine receptor

In cerebellar slices from control rats, glycine increases intracellular Cl^−^ in Purkinje cells and both cGMP and strychnine prevent the glycine-induced Cl^−^ increase (Fig. 5C). This confirms that extracellular cGMP reduces activation of glycine receptors.

**Figure 6:**
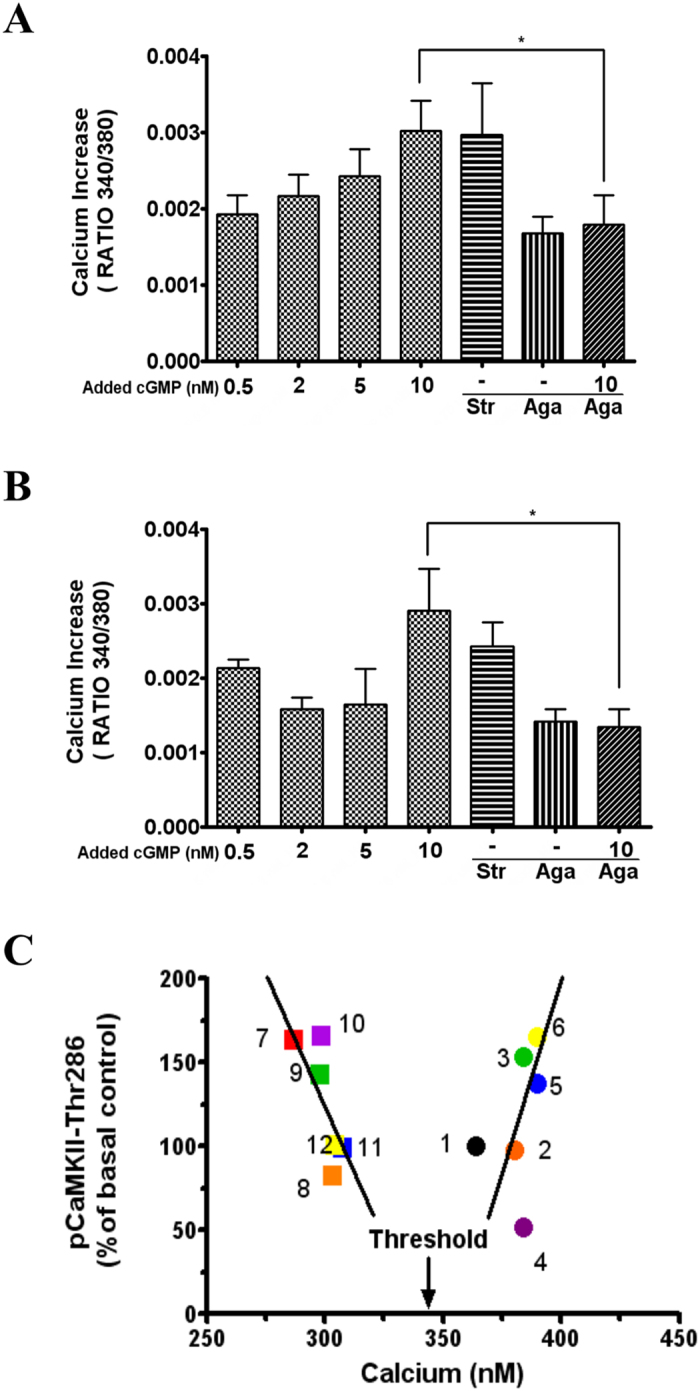
Extracellular cGMP increases intracellular calcium in cerebellar slices, which modulates CaMKII phosphorylation in a biphasic way. (**A**) Calcium increase (fluorescence ratio 340/380) in Purkinje cells in slices from control rats after addition of extracellular cGMP at the indicated concentrations; strychnine (75 μM), ω-Agatoxin IVA (10 nM) alone or in combination with 10 nM cGMP. (**B**) Shows the same data for hyperammonemic rats. Values are the mean ± SEM of at least 90 Purkinje neurons in at least 6 different slices. Values significantly different are indicated with asterisk, *(p < 0.05). **(C)** Shows the effects of cGMP or strychnine on calcium levels (in nM) and on CaMKII phosphorylation in Thr286. Values are expressed as percentage of CaMKII phosphorylation in slices from control rats under basal conditions. Data from control rats are indicated with a circle ○ and hyperammonemic rats with a square □. The different numbers indicate: 1 and 7, basal; 2 and 8, cGMP 0.5 nM; 3 and 9, cGMP 2 nM; 4 and 10, cGMP 5 nM; 5 and 11, cGMP 10 nM; 6 and 12, strychnine 75 μM. Each value is the mean of at least 9 determinations.

As CaMKII is modulated by calcium, we assessed whether modulation of glycine receptors by cGMP is associated with changes in intracellular calcium. Adding different extracellular cGMP concentrations to cerebellar slices from control rats induces a progressive increase in intracellular calcium in Purkinje neurons. Adding strychnine induced a calcium increase similar to that induced by 10 nM cGMP (Fig. 6A), supporting that the cGMP-induced calcium increase is mediated by inhibition of glycine receptors.

As glycine receptors modulate intracellular Cl^−^, we hypothesized that their effects on calcium would be mediated by voltage-dependent calcium channels. We tested whether blocking voltage-dependent calcium channels with ω-agatoxin prevents cGMP-induced increase in calcium. As shows Fig. 6A this was the case, adding 10 nM cGMP increased the 340/380 ratio by 0.003; ω-agatoxin IVA alone increased it by 0.00167 and 10 nM cGMP + ω-agatoxin IVA by 0.00179, indicating that ω-agatoxin IVA completely prevents the cGMP-induced calcium increase. This indicates that cGMP-induced calcium increase is mediated by glycine receptors inhibition and modulation of voltage-dependent calcium channels.

In Purkinje cells in cerebellar slices from hyperammonemic rats the basal intracellular calcium concentration (289 ± 20 nM) was lower (p < 0.05) than in control rats (366 ± 11 nM). The effects on intracellular calcium of extracellular cGMP were similar in hyperammonemic and control rats and mediated by the same mechanisms (Fig. 6B).

### Extracellular cGMP-induced changes in intracellular calcium modulate CaMKII phosphorylation in a biphasic way

The effects of treatments with extracellular cGMP or strychnine on calcium levels and CaMKII phosphorylation are shown in Fig. 6C.

The lowest calcium concentration was found in slices from hyperammonemic rats under basal conditions (289 nM) and was associated with a high CaMKII phosphorylation (164% of basal in controls, point 7 in Fig. 6C). Treating slices from hyperammonemic rats with different extracellular cGMP concentrations or with strychnine progressively increased intracellular calcium, reaching 304 and 308 nM for strychnine or 10 nM cGMP, respectively (points 12 and 11 in Fig. 6C).

The progressive increase in intracellular calcium in slices from hyperammonemic rats was associated with a progressive reduction in CaMKII phosphorylation, which decreased to 101 and 99% of basal in controls (returning to normal levels) for strychnine or 10 nM cGMP, respectively (points 12 and 11 in Fig. 6C).

Basal calcium levels in controls were higher (364 nM, point 1 in Fig. 6C) than in hyperammonemic rats after any treatment. Treating slices from controls rats with extracellular cGMP or strychnine increased progressively intracellular calcium, reaching 390 nM for slices treated with strychnine or 10 nM cGMP (points 5 and 6 in Fig. 6C). In contrast to hyperammonemic rats, in control rats the progressive increase in intracellular calcium was associated with a progressive increase in CaMKII phosphorylation, reaching 166 and 146% of basal for strychnine or 10 nM cGMP, respectively (points 5 and 6 in Fig. 6C). Figure 6C shows that calcium modulates CaMKII phosphorylation in a biphasic way.

### Extracellular cGMP modulates the ability to learn the Y maze task in a biphasic way

**Figure 7:**
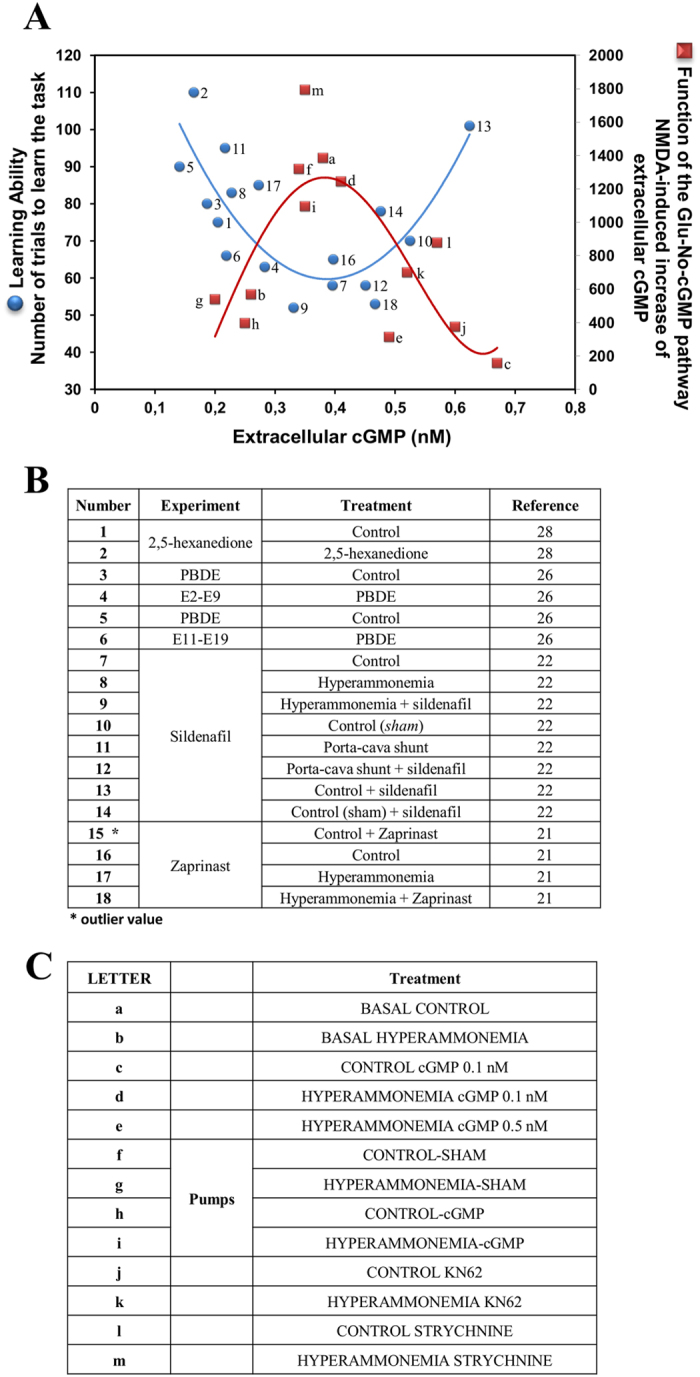
Biphasic correlations between extracellular cGMP concentration and the glutamate-NO-cGMP pathway function and ability to learn the Y maze task. (**A**) The blue curve shows the values of extracellular cGMP concentration and the ability to learn the conditional discrimination task in the Y-maze obtained in our group in different studies during last years as summarized in (**B**). The red curve shows the data on the pathway function at different concentrations of extracellular cGMP reported in the present work, as indicated in (**C**). (**B**) shows the experiments and treatments from which each point of the blue line in (**A**) has been obtained and the corresponding refs 21,22,26,28. (**C**) shows the treatments of this work from which each point of the red line in (**A**) has been obtained. Points j and k were obtained in similar experiments using KN62.

To assess whether there is a correlation between extracellular cGMP concentration and the ability to learn the conditional discrimination task in the Y-maze, we represented in Fig. 7, in blue, the values for extracellular cGMP and learning ability obtained in our group in different studies during the last years. Rats were treated with different substances that increased or decreased extracellular cGMP in cerebellum and the ability to learn the Y maze task. The blue line in Fig. 7 shows that there is a biphasic correlation between extracellular cGMP in cerebellum and the ability to learn the Y maze task. There is an optimal range of extracellular cGMP, between 0.35–0.45 nM approximately, at which learning ability is maximal, requiring fewer trials to learn. Lower or higher cGMP concentrations result in reduced learning ability.

We superimposed in Fig. 7, in red, the data on the pathway function at different extracellular cGMP concentrations. Figure 7 corroborates that the optimal ranges of extracellular cGMP for learning and for the function of the glutamate-NO-cGMP pathway are exactly the same.

## Discussion

**Figure 8:**
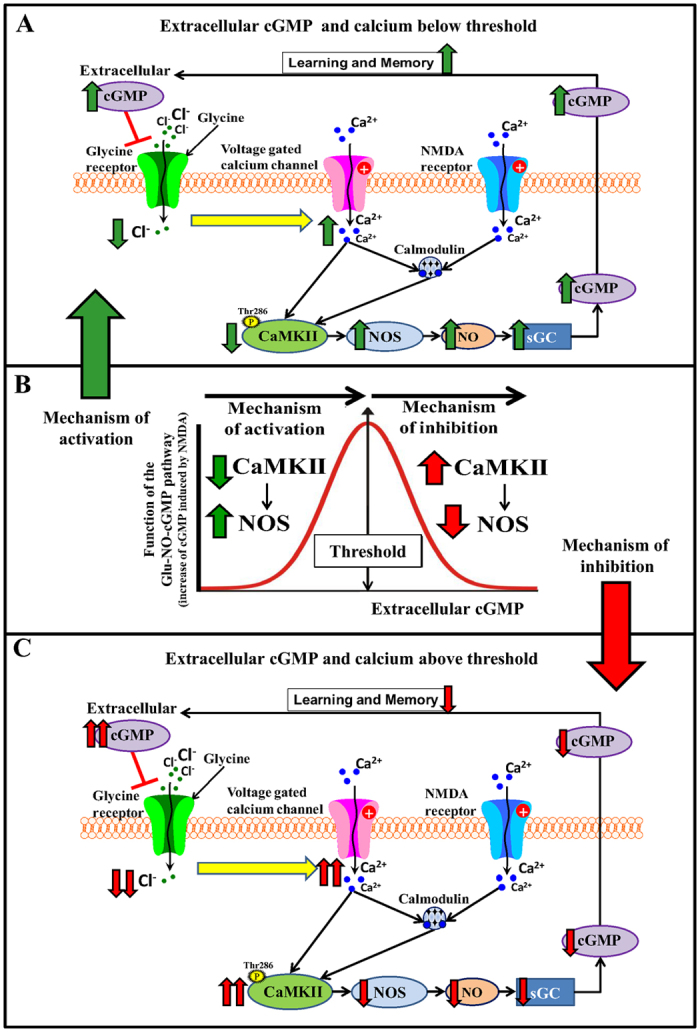
Proposed model for the mechanisms involved in the biphasic modulation of the glutamate-NO-cGMP pathway and the learning ability by extracellular cGMP. (**B**) Extracellular cGMP modulates the glutamate-NO-cGMP pathway in a biphasic way. The pathway function is optimal for a narrow range of extracellular cGMP of approximately 0.30–0.45 nM. Lower or higher cGMP concentrations result in reduced function of the pathway. (**A**) When extracellular cGMP is below the threshold indicated in (**B**), phosphorylation of CaMKII is high, reducing the function of the pathway. Addition of extracellular cGMP reduces CaMKII phosphorylation and enhances the pathway function. (**C**) When extracellular cGMP is above the threshold indicated in (**B**), phosphorylation of CaMKII is low, allowing a good function of the pathway. Addition of extracellular cGMP increases CaMKII phosphorylation and reduces the pathway function. Both mechanisms of activation (**A**) and inhibition (**B**) induced by extracellular cGMP are mediated by reducing activation of glycine receptors, which mildly increases intracellular calcium through voltage-dependent calcium channels. The modulation of the pathway by cGMP is biphasic because the increase in calcium modulates CaMKII phosphorylation in a biphasic way depending on the basal levels of calcium.

This work provides some new ideas that may help to better understand the modulation of some types of learning and how to improve them in pathological situations. It is shown that extracellular cGMP, at physiological concentrations, modulates the glutamate-NO-cGMP pathway (the response to NMDA) in cerebellum *in vivo* in a biphasic manner. In Fig. 8, we propose a model with putative mechanisms involved in this biphasic modulation. There is a narrow optimal range of extracellular cGMP (0.30–0.45 nM) for which the function of the glutamate-NO-cGMP pathway is maximal. The function is lower at higher or lower extracellular cGMP concentrations. Modulation of the pathway by extracellular cGMP is biphasic, there is a threshold (around 0.45 nM) below which increasing extracellular cGMP enhances the function of the pathway while at cGMP higher than the threshold cGMP reduces the function of the pathway (Fig. 8B).

The concentration of extracellular cGMP in normal rats is nearly the maximal concentration for optimal function of the pathway but very low increases of extracellular cGMP may still increase the function of the pathway. This occurs for example in rats perinatally exposed to PBDE99. When these rats are 2–3 months old the extracellular cGMP was slightly increased and the function of the pathway and learning ability were also increased^26^.

In pathological situations in which reduced extracellular cGMP concentration is associated with reduced function of the glutamate-NO-cGMP pathway, it is possible to recover the function of the pathway and learning ability using treatments that increase cGMP levels such as inhibitors of phosphodiesterase 5^21^,^22^.

To identify the mechanisms by which low extracellular cGMP levels activate the glutamate-NO-cGMP pathway while high concentrations inhibit it we assessed in fresh cerebellar slices the effects of adding extracellular cGMP on NOS and CaMKII activities. In hyperammonemic rats, the pathway function is reduced mainly because NOS activity is reduced due to increased phosphorylation by CaMKII, which phosphorylation and activity is increased in hyperammonemia^36^. Taking this into account, we analyzed whether extracellular cGMP modulates the pathway by modulating NOS and/or CaMKII activities. The results indicate that the activating mechanism by which low extracellular cGMP levels enhance the pathway in hyperammonemic rats involves reduction of CaMKII and enhancement of NOS activities while the inhibitory mechanism by which high extracellular cGMP levels reduce the pathway function involves enhancement of CaMKII and reduction of NOS activities (Fig. 8B).

To modulate intracellular CaMKII and NOS activities extracellular cGMP should act on some molecule in the cell membrane which would transduce the signal inside the neuron. Extracellular cGMP, at physiological (nM) concentrations, modulates glycine receptors, reducing its activation by glycine^37^. We show that strychnine, a glycine receptor antagonist, induces similar effects that cGMP on the glutamate-NO-cGMP pathway *in vivo* and on NOS and CaMKII activities in cerebellar slices.

These results support that a main molecular target for extracellular cGMP in the cell membrane is the glycine receptor, which mediates its effects on the glutamate-NO-cGMP pathway. Extracellular cGMP reduces the function of glycine receptors, reducing the increase in intracellular Cl^−^ induced by glycine in Purkinje neurons similarly to the glycine receptor antagonist strychnine. This finding is in agreement with a recent report from Bukanova *et al.*^37^ showing that, in isolated rat hippocampal pyramidal neurons, cGMP at physiological (nM) concentrations reduces the function of glycine receptors by accelerating desensitization of glycine-induced chloride currents. We show, for the first time, that modulation of glycine receptors by extracellular cGMP has functional consequences both on the activity of the glutamate-NO-cGMP pathway and on modulation of learning ability.

The modulation of the glutamate-NO-cGMP pathway mediated by glycine receptors is associated with an extracellular cGMP-induced mild increase in intracellular calcium (Fig. 8A,C) in Purkinje neurons which is prevented by blocking voltage-dependent calcium channels. As far as we know this is the first report showing that extracellular cGMP, through modulation of glycine receptors and voltage-dependent calcium channels, affects intracellular calcium levels in Purkinje cells. This supports the idea that extracellular cGMP may modulate intracellular calcium, at least to a mild extent, by reducing activation of glycine receptors and subsequent modulation of voltage-dependent calcium channels. This modulation of intracellular calcium by extracellular cGMP may affect different intracellular signaling pathways, as shown here for CaMKII and the glutamate-NO-cGMP pathway, neurotransmission and learning.

A main finding of this work is that the increase in calcium induced by extracellular cGMP modulates CaMKII phosphorylation (and activity) in a biphasic way. Under normal conditions (Fig. 8C), the increase in calcium over basal levels enhances phosphorylation of CaMKII, its activity and phosphorylation of neuronal NOS, resulting in reduced NOS activity and function of the glutamate-NO-cGMP pathway and reduced ability to learn the Y maze task. However, when calcium levels are low (Fig. 8A), below the threshold indicated in Fig. 6C, as occurs for example in hyperammonemia, phosphorylation of CaMKII is high, and the increase in calcium reduces phosphorylation of CaMKII, its activity and phosphorylation of neuronal NOS, resulting in increased NOS activity and function of the glutamate-NO-cGMP pathway and improvement of the ability to learn the Y maze task.

CaMKII phosphorylation and activity in Purkinje neurons is modulated by a very complex mechanism involving calcium levels, auto-phosphorylation of CaMKII, dephosphorylation of CaMKII by protein phosphatase 1 (PP1), inhibition of PP1 by phosphorylated inhibitor 1, and dephosphorylation (and inactivation) of inhibitor 1 by the calcium-calmodulin-dependent phosphatase calcineurin. The results reported here fit perfectly within the bistable synapse mathematical model proposed by Graupner and Brunel^38^ to explain modulation of CaMKII. This model shows that there are two stable states of phosphorylation of CaMKII at resting intracellular calcium concentrations one weakly phosphorylated (control rats in our case) and one highly phosphorylated (hyperammonemic rats in our case). The reason for this biphasic modulation of CaMKII phosphorylation by calcium is that the activity of PP1 would be maximal between 0.22 and 0.4 μM calcium (Figs 3B and 8A of ref. 38), thus reducing CaMKII phosphorylation. High calcium transients switch the system from the weakly to the highly phosphorylated form (as occurs in our case for control rats). Intermediate calcium concentrations increase CaMKII dephosphorylation (as occurs in our case for hyperammonemic rats). This model would explain therefore the biphasic effect of extracellular cGMP on CaMKII activity reported here.

The biphasic modulation of CaMKII by extracellular cGMP leads to the biphasic modulation of NOS activity and of the glutamate-NO-cGMP pathway which in turn leads to a biphasic modulation of the ability to learn the Y maze task (Fig. 8). There is a threshold (around 0.45 nM) below which increasing extracellular cGMP enhances learning ability while at cGMP higher than the threshold increasing cGMP reduces learning ability (Fig. 7). The optimal ranges of extracellular cGMP for which the function of the pathway and learning ability are maximal are exactly the same (around 0.34–0.45 nM) further supporting that the ability to learn this Y maze task is modulated by the glutamate-NO-cGMP pathway in cerebellum.

In summary, it is shown for the first time that extracellular cGMP at physiological concentrations reduces activation of glycine receptors leading to a mild voltage-dependent calcium-channels-mediated increase in intracellular calcium in Purkinje neurons. This increase in calcium modulates CaMKII phosphorylation in a biphasic way. When extracellular cGMP and basal calcium concentrations are low (as in hyperammonemia) increasing extracellular cGMP reduces phosphorylation of CaMKII, increasing NOS activity, the function of the glutamate-NO-cGMP pathway and learning ability. When extracellular cGMP and basal calcium concentration are normal extracellular cGMP increases phosphorylation of CaMKII, reducing NOS activity, the function of the glutamate-NO-cGMP pathway and learning ability.

These data unveil new mechanisms, molecules and pathways involved in modulation of the ability to learn the Y maze task and likely other types of learning and memory which may be also therapeutic targets to improve learning in pathological situations such as hyperammonemia, hepatic encephalopathy or other situations associated with low extracellular levels of cGMP.

## Methods

### Model of chronic hyperammonemia in rats

Male Wistar rats (120–140 g) were made hyperammonemic by feeding them an ammonium-containing diet as in ref. 31. The experiments were approved by the Comité de Ética de Experimentación Animal (CEEA) of the Center and by the Dirección General de Agricultura, Ganadería y Pesca, Consellería de Agricultura, Generalitat Valenciana and carried out in accordance with the European Communities Council Directive (86/609/EEC).

### Continuous intracerebral administration of cGMP to rats using osmotic pumps

Rats were divided in four groups, two of control rats and two of hyperammonemic rats. For one group of control rats and one of hyperammonemic rats, the osmotic pumps were filled with 240 μM cGMP in sterile saline. For the other two groups with the vehicle solution, sterile saline. The pumps were implanted in the back of the rats two weeks after starting the ammonium-diet. These pumps released 0.25 μl per hour during 28 days and were connected to a cannula implanted in the cerebral ventricle as in ref. 21. At the same time, a microdialysis guide was implanted in the cerebellum as described below to allow determination of extracellular cGMP.

### Analysis of extracellular cGMP and of the glutamate-nitric oxide-cGMP pathway in cerebellum by *in vivo* brain microdialysis

Rats were anesthetized using isoflurane and microdialysis was performed in cerebellum as in ref. 39. When indicated, NMDA (0.5 mM) was administered to activate the glutamate-NO-cGMP pathway. Samples were made 4 mM in Ethylenediaminetetraacetic acid (EDTA) and stored at −80 °C until analysis of cGMP. Cyclic GMP was determined with the BIOTRAK cGMP enzyme immunoassay kit from Amersham (Amersham Biotrak GE Healthcare) using 50 μL of dialysate from cerebellar microdialysis experiments.

To assess the effects of extracellular cGMP, several concentrations of cGMP (in nM) were administered through the microdialysis probe. After a 2–2.5 h period, NMDA was administered to assess the effects of administration of extracellular cGMP on the activation of the glutamate-nitric oxide-cGMP pathway. Extracellular cGMP was measured as described above. To assess the effects of the glycine receptor inhibition on the activation of the pathway, strychnine (4 μM) was administered through the microdialysis probe during 2,5 h and after NMDA. To assess the effects of the CaMKII inhibition on the activation of the pathway, KN62 (1 μM) was administered through the microdialysis probe during 2,5 h and after NMDA. Extracellular cGMP was measured as described above.

To quantify the function of the glutamate-NO-cGMP pathway, we measured the response to NMDA: the increase in extracellular cGMP induced by NMDA in the 4 fractions after its administration was calculated and expressed as percentage of basal levels.

### Analysis of the activation of nitric oxide synthase in cerebellum slices

Rats were sacrificed by decapitation and cerebellar slices were prepared as in ref. 36. cGMP (10 nM) and strychnine (75 μM) were added and incubated for 20 min. After the treatment, slices were homogenized and the activation of NOS was determined by the conversion of [^14^C] L-Arginine to [^14^C] citrulline^40^. NOS activity is expressed as the difference between [^14^C]citrulline formed in absence and presence of nitroarginine.

### Analysis of the phosphorylation of CaMK-II in Thr286

Cerebellar slices were prepared and treated with cGMP and strychnine, as described above. Slices were collected and homogenized in a buffer (Tris-HCl 66 mM pH 7.4, SDS 1%, EGTA 1 mM, Glycerol 10%, Leupeptin 0.2 mg/mL, NaF 1 mM, Na orto-vanadate 1 mM). Samples were subjected to immunoblotting as in ref. 41 using antibodies against CaMKII phosphorylated at Thr 286 (1:1000) and CaMKII (1:2000) from abCam (Cambridge, UK). The band intensities were quantified using the Alpha Imager 2200 program (AlphaEaseFC 2200 for Windows, Cambridge, UK).

### Analysis of intracellular chloride and calcium dynamics in living cerebellum slices using fluorescence microscopy

To measure intracellular chloride in cerebellum slices, 6-Methoxy-N-Ethylquinolinium Iodide (MEQ) (Molecular Probes, Eugene, OR) was reduced to its cell-permeable derivative dihydro-MEQ before bathloading slices, as described by Schwartz and Yu^42^. To measure intracellular calcium we used the probe Fura-2AM (Molecular Probes). For all experiments, slices were allowed to equilibrate in the chamber for at least 15 min. Then slices were incubated during 30 min with the probe and washed with Krebs buffer perfused for at least 15 min before the baseline recording period. The incubation was performed in the dark and the slices were kept in constantly oxygenated buffer in the dark until placed in the imaging chamber. Dye-loaded slices were transferred to an imaging chamber on an upright microscope (Leica DM2500) and constantly perfused with oxygenated Krebs buffer at room temperature. Fluorescence (brightness intensity) was measured through a 20 X water-immersion objective (HCX APO L20X/0.5W U-V-I, Leica) with a 344 nm excitation filter, a 442 nm emission filter for dihydro-MEQ or 340/380 nM excitation ratio for fura-2. Fluorescence emission was detected with a camera (DFC360FX, Leica Microsystems). Emission data were collected and stored on disk with the Image Analysis Program (Leica LAS AF). To measure intracellular chloride, approximately 5 min after the slices were placed in the imaging chamber, glycine alone or in combination with cGMP or Strychnine was perfused to the chamber and the change (decrease) in fluorescence was recorded in Purkinje and granular cells. To measure intracellular calcium, several concentrations of extracellular cGMP or Strychnine or ω-Agatoxin IVA (selective blocker of P-type calcium channels) alone or in combination with cGMP was perfused to the chamber and the change (increase) in fluorescence was recorded in Purkinje cells. At least six rats were used to collect data for each experiment.

### Determination of calcium concentration

Calcium concentration was calculated according to Bootman *et al.*^43^ using the equation: [Ca^2+^](nM) = Kd*(F^380^max/F^380^min)*(R-Rmin)/(Rmax-R). R is the ratio of emission intensity at 510 nm with excitation at 340 nm, to emission intensity at 510 nm with excitation at 380 nm; Rmin is the ratio at zero free Ca^2+^; Rmax is the ratio at saturating Ca^2+^ (20 mM); F^380^max is the fluorescence intensity with excitation at 380 nm, for zero free Ca^2+^; F^380^min is the fluorescence intensity at saturating free Ca^2+^.

F^380^max were measured in slices using a solution of Krebs buffer + 20 μM ionomycin + 5 mM ethylene glycol-bis(β-aminoethyl ether)-N,N,N’,N’-tetraacetic acid (EGTA), without calcium. F^380^min were measured in slices using a solution of krebs buffer + 20 μM ionomycin + 20 mM calcium. The constant used in this equation is: Kd = 230, which is the value for Fura-2AM at the temperature and pH conditions used in these experiments. The other values were: Fmax/Fmin = 1.75; Rmin = 0.0385 and Rmax 0.2154. The value of R is the mean of the ratio F340/F380 for at least 60 neurons of different slices.

### Data analysis for MEQ fluorescence imaging

Because MEQ fluorescence is quenched collisionally by Cl^−^, increases in intracellular Cl^−^ correspond directly with a decrease in fluorescence. MEQ is not a ratiometric indicator, therefore absolute intracellular concentrations of Cl^−^ were not measured. The change in intracellular Cl^−^ was expressed as the change in MEQ fluorescence (ΔF) of Purkinje cells compared to granular cells, as described by Inglefield and Schwartz-Bloom^44^.

### Learning of a Conditional Discrimination Task in a Y-Maze

Learning ability was tested 1 week after implantation of osmotic pumps as in Aguilar *et al.*^45^ in a Y-shaped maze. The colour of the walls may be white or black. Rats must learn where is the food depending on the colour of the walls. Rats performed 10 trials per day, until the completion of a criterion of 10 correct responses in the same day or a maximum of 250 trials.

## Additional Information

**How to cite this article**: Cabrera-Pastor, A. *et al.* Extracellular cGMP Modulates Learning Biphasically by Modulating Glycine Receptors, CaMKII and Glutamate-Nitric Oxide-cGMP Pathway. *Sci. Rep.* **6**, 33124; doi: 10.1038/srep33124 (2016).
